# Hybridization and emergence of virulence in opportunistic human yeast pathogens

**DOI:** 10.1002/yea.3242

**Published:** 2017-09-14

**Authors:** Verónica Mixão, Toni Gabaldón

**Affiliations:** ^1^ Centre for Genomic Regulation The Barcelona Institute of Science and Technology Dr. Aiguader 88 Barcelona 08003 Spain; ^2^ Universitat Pompeu Fabra 08003 Barcelona Spain; ^3^ Institució Catalana de Recerca i Estudis Avançats Pg. Lluís Companys 23 08010 Barcelona Spain

**Keywords:** hybridization, yeast, comparative genomics, pathogens, emergence of virulence

## Abstract

Hybridization between different species can result in the emergence of new lineages and adaptive phenotypes. Occasionally, hybridization in fungal organisms can drive the appearance of opportunistic lifestyles or shifts to new hosts, resulting in the emergence of novel pathogens. In recent years, an increasing number of studies have documented the existence of hybrids in diverse yeast clades, including some comprising human pathogens. Comparative and population genomics studies performed on these clades are enabling us to understand what roles hybridization may play in the evolution and emergence of a virulence potential towards humans. Here we survey recent genomic studies on several yeast pathogenic clades where hybrids have been identified, and discuss the broader implications of hybridization in the evolution and emergence of pathogenic lineages. © 2017 The Authors. *Yeast* published by John Wiley & Sons, Ltd.

## Introduction

Inter‐species hybrids result from the crossing of two diverged species. Hybrids are thus chimeric organisms that carry material from two differentiated genomes and may display a range of properties present in either of the two parent lineages, as well as novel, emerging phenotypes that differentiate them from both of their parents. As such, hybridization has been long recognized as an important evolutionary mechanism that can drive new adaptive phenotypes that enable the colonization of new environments (Gladieux et al., 2014). Studies on hybridization have been traditionally performed on animals and plants, where hybrids are easy to recognize from their morphology. Such studies have established hybridization as an important evolutionary force driving the origin of new lineages and important ecological adaptations (Fonseca et al., 2004; Lee et al., 2013; Lunt et al., 2014; Masuelli et al., 2009; Session et al., 2016). In fungi, where hybrids are difficult to recognize morphologically or physiologically, the study of hybrids has long been neglected. However, the advent of genomics has recently enabled the identification of a growing number of fungal hybrids belonging to diverse clades and among industrial, clinical or environmental strains (Leducq et al., 2016; Morales & Dujon, 2012; Pryszcz et al., 2014; Pryszcz et al., 2015). Although most identified hybrids are thought to have been formed relatively recently, hybridization may have played an important role in the origin of some ancient lineages. Indeed, hybridization has recently been recognized as the process underlying the major whole genome duplication that occurred around 100 million years ago in the lineage leading to *Saccharomyces cerevisiae* (Marcet‐Houben & Gabaldón, 2015).

Besides its ecological and evolutionary importance, the study of hybridization is important from a physiological perspective. The situation created by the merging of two genomes, and the resulting transcriptomes and proteomes within a single cell, has been described as a ‘genomic shock’ (McClintock, 1984). In particular, at the early stages, physiological processes in hybrid organisms are expected to be intrinsically unfit, owing to interferences in the cross‐talk of two genetic systems that have evolved separately for some time. Thus, even if some of the emerging new properties in the hybrid ensure a selective advantage in a given niche, the hybrid fitness will benefit from purging existing deleterious interactions between the two sub‐genomes. This process is referred to as genome stabilization, and is mediated by several mechanisms including genomic recombination, gene conversion, or chromosomal loss or duplication (Payseur & Rieseberg, 2016). Although events mediated by such mechanisms have a stochastic mutational source, they can be subject to selection. Thus, evolution of fungal hybrid lineages follows particular rules, and we currently lack sufficient understanding of its tempo and mode.

In the last decade, the incidence of fungal infections has increased, partly owing to recent advances in the medical sector such as the increased survival of immunocompromised patients, the extensive use of antibiotics, immunosupressors or medical devices such as catheters (Gabaldón & Carreté, 2016; Pfaller & Diekema, 2007). This increase in incidence is due not only to a higher number of reported cases caused by known pathogenic species, but also an increasing number of infections whose underlying cause is a rare species (Pfaller & Diekema, 2004). For instance, among the etiological causes of invasive candidiasis, there are over 30 different species (Gabaldón et al., 2016), of which more than half can be considered very rare (i.e. <0.1% of the cases). This has led to the concept of emerging fungal pathogens (Papon et al., 2013). Although the above‐mentioned progress in the medical sector is certainly one of the factors driving the increase in incidence and number of etiological agents, it is unclear whether other factors such as species dispersion to new environments may play a role. In addition, although the number of fungal species causing infection is increasing, there are clear differences among them in terms of their ability to cause infection and damage to the human host. In summary, we still have a very poor understanding of the genomic properties that may underlie a virulent outcome between a potential pathogen and its host, and how they have emerged during evolution.

In recent years hybridization has emerged as a potential important source of new pathogenic species. Indeed, a growing number of studies report the presence of hybrids among clinical isolates, in particular in yeast clades such as *Cryptococcus* or *Candida* (Boekhout et al., 2001; Pryszcz et al., 2014; Pryszcz et al., 2015; Schroder, 2016; Viviani et al., 2006). These findings are worrisome, particularly considering current processes such as global trade, environment alteration and climate change, which may be favouring the encounter of divergent species that can still hybridize. The sources and potential implications of hybrids among clinical isolates are still poorly understood, and hence there is a need for studying the distribution, evolution and physiology of pathogenic hybrids. In this review we survey current knowledge about hybrids in opportunistic human pathogens, with a special emphasis on their genomic features and the mechanisms underlying their evolution. The possible relationship between hybridization events and the development of virulence will also be discussed.

## Hybridization and the origin of emerging phenotypes

Hybrids are chimeric organisms carrying two different genomes that must coexist within the same cell. This phenomenon does not occur frequently in nature because on many occasions the chimeric organism is simply not viable. In the first instance mating between two organisms may be prevented by physiological pre‐zygotic barriers such as problems in gamete recognition. If mating is possible and a zygote is formed, numerous post‐zygotic barriers can exist. For instance, developmental problems may arise, aborting the generation of a new individual or, if the hybrid is viable, it may not be able to reproduce. Ultimately, the hybrid will need to survive in competition with other species. Certainly, all natural hybrid species that we now recognize are a small fraction of those that were initially formed, and they all must present some characteristics that promoted their survival.

Hybridization events can promote the origin of extreme phenotypes and adaptations to new ecological environments (Gladieux et al., 2014). This adaptation to new environments is key for hybrids' survival, as it not only allows isolation from the parent species but also it may provide a competitive advantage that allows for a relatively high fitness, despite the potential deleterious effects of the underlying ‘genomic shock’ (see below). Adaptation to new niches is generally promoted by the presence in the hybrid of transgressive phenotypes, that is, those phenotypes or characteristics that are beyond those present in the parent species. The sunflower *Helianthus paradoxus*, a hybrid between *Helianthus annuus* and *H. petiolaris* is an example of this, as it can colonize soils with a high concentration of salt, where neither of the two parents can survive (Welch & Rieseberg, 2002a; Welch & Rieseberg, 2002b). This possible existence of a superior performance of hybrids when compared with their parent lineages was described in 1908 by Shull, who later named this characteristic ‘heterosis’ (Shull, 1908; Shull, 1914). Heterosis can result from multiple mechanisms (Lippman & Zamir, 2007; Swanson‐Wagner et al., 2006), and some studies point to the existence of a correlation between the parent expression levels and the hybrid performance (Frisch et al., 2010; Thiemann et al., 2010). This ability of hybrids to adapt to new conditions and show transgressive phenotypes has been highly exploited in agriculture and in fact several crops that we include in our diet are hybrids (Bevan et al., 2017; Lippman & Zamir, 2007; Warschefsky et al., 2014). Besides agriculture, industries such as beer‐ and wine‐making are also taking advantage of the different characteristics of hybrid organisms (Pretorius & Bauer, 2002). *Saccharomyces pastorianus* is a hybrid yeast which keeps the strong fermenting ability of its parent *S. cerevisa*e as well as the ability to survive under low temperatures of its parent *S. eubayanus* (Gibson & Liti, 2015). These two characteristics are essential for lager beer production, and therefore this organism has had an advantage compared with each of the parents, being widely used in this industry. Similarly, several hybrids between *S. cerevisiae* and other *Saccharomyces* species such as *S. kudriavzevii* present the ability to grow under ethanol and temperature stress (Belloch et al., 2009). This characteristic has been extensively explored by wine‐makers, who prefer to produce white wine under low temperatures, minimizing the loss of aromatic compounds, which makes these hybrids an important economic asset in this business (reviewed in Marsit and Dequin (Marsit & Dequin, 2015)). These are two of many possible examples of the relevance of hybridization for our daily life. In fact, these industries are increasingly recognizing the high potential of hybrids in adapting to new conditions and developing advantageous phenotypes. Currently, the artificial generation of new hybrids with useful characteristics in the laboratory constitutes a promising innovation strategy (Krogerus et al., 2017).

How can hybrids achieve these advantages? In crops, the mechanisms underlying heterosis have been extensively studied, and gene expression changes were reported in intra‐specific hybrids of rice, maize and wheat (Guo et al., 2006; He et al., 2010; Stupar & Springer, 2006; Swanson‐Wagner et al., 2006; Wang et al., 2006). These changes can be additive or non‐additive when, respectively, the value of gene expression is the mean of both parent lineages, or the expression of one parent is decreased while the other is increased (reviewed in Chen (Chen, 2013)). However, despite all of the studies performed to understand these mechanisms, the underlying molecular basis of heterosis is still poorly understood. More studies are definitely needed in order to understand this question better.

## Genomic impacts of hybridization

**Figure 1:**
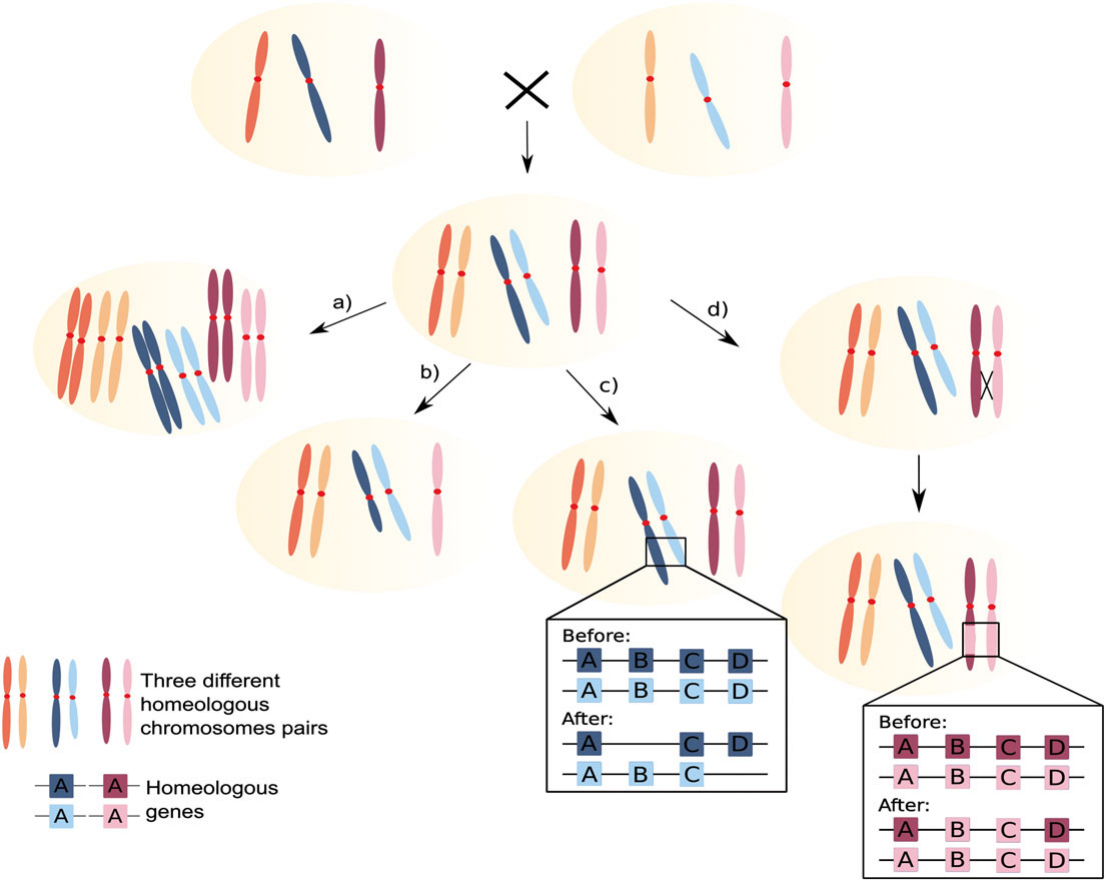
Schematic representation of several different mechanisms that can lead to genome stabilization in hybrids. Shaded ovals represent cells. Chromosomes are painted in different colours with different tones of the same colour indicating homeologous pairs of chromosomes. In some cases insets highlight specific regions with genes indicated as coloured boxes. From top to bottom, two haploid cells from different species cross and form a diploid hybrid. Four non‐exclusive, alternative evolutionary paths to genome stability are shown: (a) whole‐genome duplication; (b) total or partial chromosome loss; (c) gene loss; and (d) gene conversion and loss of heterozygosity

As chimeric organisms, hybrids combine the genetic material of the two parent lineages. This implies a certain degree of genetic divergence between the homeologous chromosomes, that will initially equal the divergence between the two parent species at the time of hybridization. The resulting high levels of genetic heterozygosity may directly impact the functioning of the cell. Indeed, as suggested by the Bateson–Dobzhansky–Muller model (Bateson, 1909; Dobzhansky, 1934; Muller, 1942), different proteins for the same biological process can be produced at the same time, generating some incompatibilities that can influence the survival or fertility of the organism. Therefore, selection will favour any change that removes negative epistatic interactions in these heterozygous genomes, thereby increasing the chances of survival. In addition, even in the absence of selection, genomic changes involving differential loss of chromosomal regions or gene conversion will inevitably result in loss of heterozygosity (LOH) in particular regions. The combination of these selective and neutral processes will result in a progressive shaping of the heterozygous genome, which would gradually lose heterozygosity, while increasing its genomic stability. Several processes can contribute to this stabilization (Figure 1). For instance, the duplication of the entire set of chromosomes through whole genome duplication would restore proper pairing between chromosomes, thus restoring the ability to go through meiosis (Marcet‐Houben & Gabaldón, 2015; Wolfe, 2015). Alternatively duplication or loss of individual chromosomes, leading to chromosomal aneuploidies, can also enhance genomic stability (Wertheimer et al., 2016). More specific mechanisms may contribute as well to this shaping process, namely gene loss, not only through literally deletion of the genomic region, but also through pseudogenization (Albalat & Cañestro, 2016), or through gene conversion, a process where a DNA sequence from one chromosome substitutes the one of its homeologue chromosomes so that the two regions become identical (McGrath et al., 2014). Events of gene loss and gene conversion lead to LOH (Figure 1). This LOH is highly associated with the evolution of hybrids (Li et al., 2012; Louis et al., 2012; Stukenbrock et al., 2012), being an extremely important mechanism leading to the reduction of genomic incompatibilities present in such organisms.

Another relevant factor for the stabilization of hybrid genomes is gene expression. Gene expression is regulated with *cis* (promoter region) and *trans* (elsewhere in the genome) elements, and in hybrid genomes it is possible that the *cis* component of one parent is regulated by the *trans* component of the other, which can alter gene regulation. As shown by Tirosh and colleagues, alterations in either *cis* or *trans* regulatory elements are important to obtain genomic stability, and therefore the reprogramming of gene expression should also be considered as an important factor in the stabilization of hybrid genomes (Tirosh et al., 2009). Furthermore, alterations in reprogramming of gene expression were shown to impact the way the hybrid interprets sensory signals (Tirosh et al., 2009). This discovery raises the question whether all of these large alterations in hybrid genomes can have consequences in the way the organism interacts with the surrounding environment. It would be interesting in future works to address the question of whether these alterations in the signal perception can drive changes in the signal response, and consequently, for example, result in the virulence of a given hybrid pathogen.

## Emerging pathogens and the evolutionary origins of virulence

**Figure 2:**
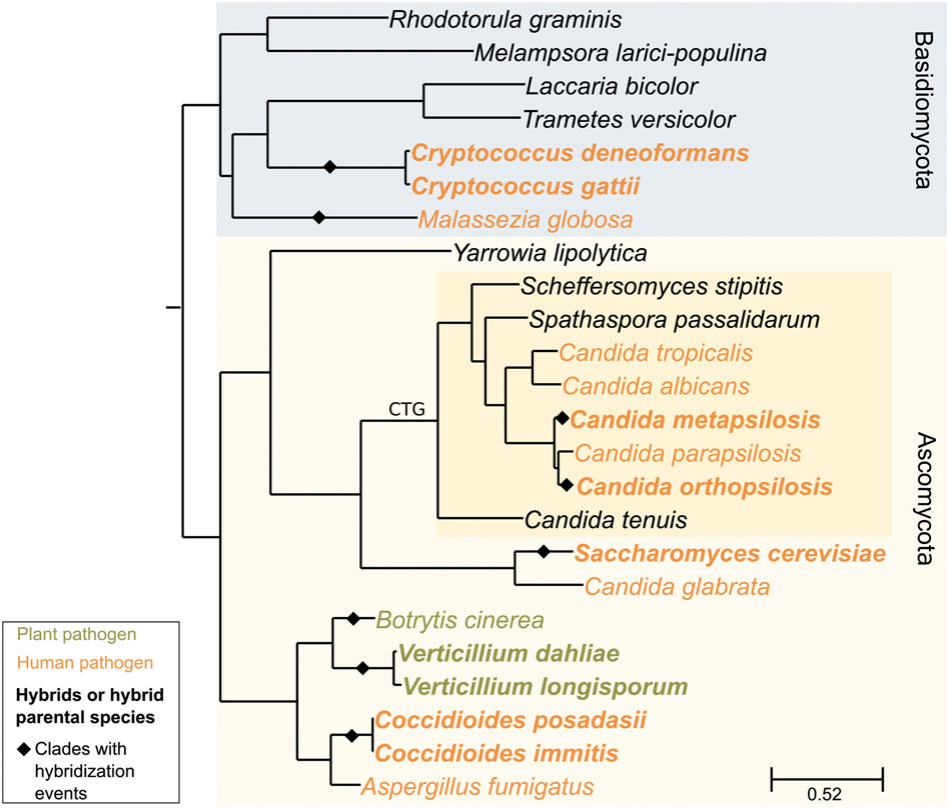
Evolutionary tree depicting the principal fungal clades with hybridization events in the origin of emergent pathogens. Clades where hybridization events have already been reported are indicated with the diamond symbol (◆). Hybrids or hybrid parent species are in bold. Basidiomycota and Ascomycota phyla are presented with blue and yellow backgrounds, respectively. Dark yellow highlights the CTG clade. Species already described as possible plant pathogens are in green, while those already described as possible human pathogens are in orange. The tree was reconstructed based on a set of four marker genes able to resolve fungal phylogenies (Capella‐Gutierrez et al., 2014). Genes were aligned and trimmed following PhylomeDB pipeline (Huerta‐Cepas et al., 2014), and the tree was reconstructed using the raxmlHPC‐PTHREADS‐SSE3 option of RAxML v8.2.4 (Stamatakis, 2014) set with PROTGAMMALG substitution model

Virulence results from the interaction of a microorganism with the host; as such it is an emerging property that depends on factors ranging from the genetic determinants of the host and the microorganism to the particular physiological or environmental conditions (Casadevall, 2012). According to Gabaldón and Carreté, not exclusively but particularly in opportunistic pathogens, virulence should be regarded as a secondary effect or an ‘evolutionary accident’, resulting from traits resulting from adaptations to selective pressure different from that involved in the pathogenic process itself (Gabaldón & Carreté, 2016). The number of fungal organisms with the ability to cause disease in humans may seem large, but it is only a small fraction of those that come into contact with us, as part of our microbiota or our environment. In addition, human fungal pathogens belong to evolutionary distinct clades and always have close relatives that are unable to infect humans (Figure 2). Thus, the ability to infect humans must have emerged several times independently. Uncovering what genomic changes may have promoted the emergence of virulence may serve to uncover novel virulence mechanisms. Despite recent efforts, we know very little about the evolution of virulence in fungi, although several trends are emerging, such as an increased cell‐wall repertoire and adherence properties in pathogens as compared with closely related non‐pathogens (Gabaldón et al., 2016). Hybridization provides an evolutionary scenario where radical genomic changes occur and where new emerging phenotypes may appear. If the new phenotype relates to the ability to survive in the human host, or an enhanced evasion from the immune system, we may obtain an increased virulence potential in a possibly newly created lineage. The relationship between hybridization processes and the development of virulence potential is not well understood. However, hybridization is being recognized as an important path to virulence in the emergence of novel plant fungal pathogens (Depotter et al., 2016), and several human pathogenic species have been shown to present hybrids, such as *Candida orthopsilosis*, *C. metapsilosis* or *Cryptococcus neoformans* (Boekhout et al., 2001; Pryszcz et al., 2014; Pryszcz et al., 2015; Schroder, 2016).

As discussed above, the initial instability present in hybrid genomes can represent a problem for these organisms as it has consequences in their fitness or survival. However, at the same time this instability results in high phenotypic plasticity and variability, which can represent an advantage in the adaptation to new environments. For example, the plant oomycete pathogen *Phytophthora* *xserendipita* is a hybrid originated through the mating of *Phytophthora cactorum* and *P. hedraiandra*, which infects the monocotyledon and dicotyledon species outside the host spectra of both parent species (Man in ‘t Veld WA, de Cock AWAM, Summerbell RC., 2007). Thus, in the context of pathogenic organisms, hybridization can have an impact on the adaptation not only to different hosts, but also to different organs, as well as to different drugs.

The globalization and mobility of goods and people all over the world can contribute to the spread of new variants or species to new geographical locations. It is known that there is less reproductive isolation between organisms that are geographically distant, and that the emergence of hybrid pathogens is often associated with the introduction of new microbes in a given area (Depotter et al., 2016). In addition, global climate change and other alterations of the environment are changing the potential geographic distribution of species, favouring the colonization of new locations and opening the possibility that previously isolated species come into contact. Therefore it can be speculated that these interchanges may contribute to the present and future increase of the emergence of hybrid species. Below, we survey recent studies on hybrids from the main clades of human pathogenic yeasts.

## *Cryptococcus neoformans* and *Cryptococcus gattii*

The *Cryptococcus neoformans*/*Cryptococcus gattii* species complex comprises a very heterogeneous sets of basidiomycete species, and its taxonomic organization has been reformulated several times during the last decade. Until the beginning of the present century, only one formal species was recognized – *C. neoformans*, with three recognized varieties, *C. neoformans var. neoformans* (corresponding to the serotype D), *C. neoformans var. grubii* (serotype A) and *C. neoformans var. gattii* (serotypes B and C). However, in 2001, the analysis of molecular markers indicated the existence of two separated species: *C. neoformans var. neoformans* and *var. grubii* and *Cryptococcus gattii* (Boekhout et al., 2001). More recently, a taxonomic reclassification based on phylogenetic analyses was proposed, and up to seven different species are now being considered. The two varieties of *C. neoformans* correspond to *C. neoformans* (previous *var. grubii*) and *C. deneoformans* (previous *var. neoformans*), and *C. gattii* was divided into five different species, namely, *C. gattii*, *C. bacillisporus*, *C. deuterogattii*, *C. tetragattii* and *C. decagattii* (Hagen et al., 2015).

All of these species can cause human cryptococcosis (Brown et al., 2012; Hagen et al., 2015). Infections from these yeasts generally start by fungal spore cells reaching the human lungs through inhalation from the environment, since their main reservoir is trees (Cogliati et al., 2016). Once in the lungs the fungus can develop into a potentially fatal infection in immunocompromised patients, particularly AIDS patients (Viviani et al., 2006). In recent years however, some *Cryptococcus* outbreaks have affected presumed immunocompetent hosts in USA, Canada and Australia (Byrnes & Heitman, 2009; Byrnes & Marr, 2011; Galanis E, MacDougall L, Kidd S, Morshed M, British Columbia Cryptococcus gattii Working Group, 2010; Pappas, 2013). From the lungs, *Cryptococcus* infections can spread to other parts of the human body, causing additional clinical complications such as meningoencephalitis (Brown et al., 2012). The number of cryptococcosis in non‐HIV patients is increasing (Bratton et al., 2012; Henao‐Martínez & Beckham, 2015) and it is estimated that this disease affects 1 million people every year, killing around 625 000 (Park et al., 2009). However, given that not all countries have data available (Viviani et al., 2006), this number could be an underestimate. In France the presence of 0.3 infections per 100 000 persons/year was reported, with a fatality rate of 15%, while in Germany, between 2004 and 2013, 491 cases were reported (Bitar et al., 2014; Smith et al., 2015).

**Figure 3:**
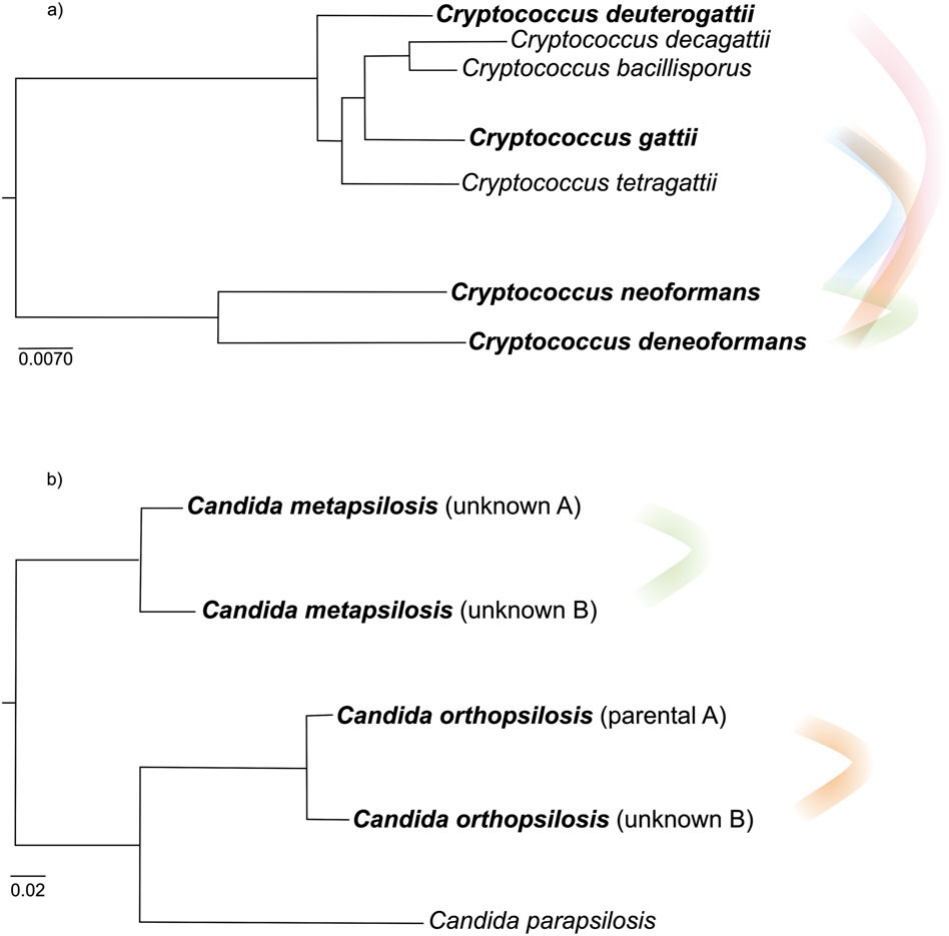
Schematic trees with representation of the different hybridization events already described in (a) *Cryptococcus* and (b) *Candida parasilosis* s.l. clades. For each tree, hybrid parent species are in bold and connected with coloured curve lines. In each tree, different colours represent different hybridization events. The tree representing *Cryptococcus* clade was adapted from Hagen *et al*. (Hagen et al., 2015), while the one representing *C. parapsilosis* clade was adapted from Pryszcz *et al*. (Pryszcz et al., 2015). [Colour figure can be viewed at wileyonlinelibrary.com]

Molecular studies on the genetic background of *Cryptococcus* samples revealed the presence of several inter‐species hybrids (Figure 3a), from which the AD hybrids (*C. neoformans* × *C. deneoformans* hybrids) are the most studied (see Table 1 for more information). The presence of different hybridization events could eventually point to a high level of similarity between the different *Cryptococcus* genomes. However, these species are highly divergent at the nucleotide level, *C. neoformans* and *C. deneoformans* having 7% divergence, and *C. gattii* and *C. deneoformans* 13% divergence. Moreover, *C. deuterogatii* has 7.6% nucleotide divergence when compared with *C. gatti*, and 14.5% when compared with *C. deneoformans* (Table 1) (D'Souza et al., 2011; Janbon et al., 2014). The presence of hybrid species in *Cryptococcus* infections is a reality, with up to 30% of all infections being associated with AD hybrids (Viviani et al., 2006). Indeed, some hybrid strains were shown to present an increased virulence, suggesting that hybridization is associated with that trait (Hagen et al., 2015; Li et al., 2012). This represents a special concern, particularly if we take into account the fact that these pathogens are already revealing some heterosis as well as resistance to antifungal drugs (Li et al., 2012). Some studies point to the existence of more than one hybridization event in the origin of some *Cryptococcus* hybrids (Li et al., 2012; Xu et al., 2002), whose genomes are experiencing continuous chromosomic changes (Li et al., 2012; Rhodes et al., 2017), possibly reflecting the above‐mentioned process of genome stabilization, but at the same time facilitating the path for pathogen evolution. Genomic analyses of AD hybrids revealed the presence of several aneuploidies, with some chromosomes from a specific parent being preferably retained (Hu et al., 2008). For instance, in chromosome 1, whose aneuploidies have already been associated with drug resistance (Sionov et al., 2010), there is a preference to retain the serotype A copy, which is sometimes duplicated, while its homeologue from serotype D is lost (Hu et al., 2008; Rhodes et al., 2017). It is notable that, for eight AD hybrid strains recently analysed, a common origin was proposed at the same time that they were shown to have undergone different evolutionary paths (Rhodes et al., 2017). This is representative of the high plasticity of these genomes, and the high diversity generated after one hybridization event, contributing to the evolution of pathogenicity in these organisms.

**Table 1:** Recognized hybrid human pathogenic yeasts, and respective parent information, as well as evidence of the occurrence of hybridization events. Columns indicate, in this order: hybrid Phylum; hybrid name; first parent species; second parent species; genomic sequence divergence at the nucleotide level between the parent species; type of analysis used for hybrid detection; and literature where this information was retrieved from

Phylum	Hybrid	Parental A	Parental B	Div	Evidence	Reference
Asco	*Candida orthopsilosis*	*Candida orthopsilosis*	Unknown	5%	Genome	(Pryszcz et al., 2014; Schroder et al., 2016)
Asco	*Candida metapsilosis*	Unknown	Unknown	4.5%	Genome	(Pryszcz et al., 2015)
Asco	*Coccidioides immitis* × *Coccidioides posadasii*	*Coccidioides immitis*	*Coccidioides posadasii*	n.a.	Genome	(Neafsey et al., 2010)
Asco	*Fusarium keratoplasticum*	*Fusarium keratoplasticum*	FSSC 9	n.a.	Markers	(Short et al., 2014; Short et al., 2013)
Basidio	*Cryptococcus neoformans* × *Cryptococcus deneoformans* (AD hybrids)	*Cryptococcus neoformans*	*Cryptococcus deneoformans*	7%	Genotype	(Boekhout et al., 2001; Janbon et al., 2014; Rhodes et al., 2017)
Basidio	*Cryptococcus deneoformans* × *Cryptococcus gattii* (BD hybrid)	*Cryptococcus deneoformans*	*Cryptococcus gattii*	13%	Genotype	(Bovers et al., 2008; D'Souza et al., 2011)
Basidio	*Cryptococcus neoformans* × *Cryptococcus gattii* (AB hybrid)	*Cryptococcus neoformans*	*Cryptococcus gattii*	n.a.	Genotype	(Bovers et al., 2008)
Basidio	*Cryptococcus neoformans* × *Cryptococcus deuterogattii*	*Cryptococcus neoformans*	*Cryptococcus deuterogattii*	n.a.	Genotype	(Aminnejad et al., 2012)
Basidio	*Malassezia furfur*	*Malassezia furfur*	*Malassezia furfur*	n.a.	Markers and genome	(Theelen et al., 2001; Wu et al., 2015)

Asco, Ascomycota; Basidio, Basidiomycota; Div, genomic divergence at nucleotide level; n.a, information not available; Genome, genomic analysis; Markers, genetic markers.

Some *Cryptococcus* pathogenic species appear as coexisting in the same niche (Cogliati et al., 2016), showing that they are constantly in contact and exposed to the possibility of occurrence of other hybridization events. The number of different hybrid species in this clade, and thus the apparent ease with which hybrids are formed recursively from independent events between the same lineages, is remarkable. This may be indicative of some special predisposition for these species to cross. This makes imperative the study of these hybrid genomes in order to understand their origin and genomic aftermath.

## *Candida parapsilosis* complex

The *Candida parapsilosis* complex comprises three recently recognized species (Figure 3b) with a worldwide distribution: *Candida parapsilosis*, *C. orthopsilosis* and *C. metapsilosis (*Tavanti et al., 2005 *)*. All of these species are facultative commensals of human skin or mucosae, and all have the ability to form biofilms in catheters and other medical devices, being associated with opportunistic nosocomial infections (Lattif et al., 2010; Melo et al., 2011; Trofa et al., 2008). Hence, these species represent a special concern for AIDS, surgery and cancer patients, and other patients with long‐term use of venous treatment. These opportunistic pathogens can be associated with episodes of fungemia, endocarditis, peritonitis, endophtalmitis, otomycosis and less frequently meningitis and vulvovaginal or urinary infections (Trofa et al., 2008). The treatment of these infections has to take into account their lower susceptibility to echinocandins as well as the possible presence of resistance to fluconazol (Garcia‐Effron et al., 2008; Lockhart et al., 2008), which constitute the usual drugs used in candidemia treatment.

The human pathogenicity observed in this clade evolved independently from that in other *Candida* spp. (Pryszcz et al., 2015). Even so, *C. parapsilosis* ranks as the third most common cause of candidiasis worldwide, after *C. albicans* and *C. glabrata*, being the *Candida* species with the highest increase in incidence in recent years (Diekema et al., 2012; Trofa et al., 2008). *C. parapsilosis* is the most prevalent of the three species of the complex, and its infections particularly affect neonates, which account for one‐third of *C. parapsilosis* infections and have a relatively high mortality rate of around 10% (Chow et al., 2012; Pammi et al., 2013). Like *C. parapsilosis*, *C. orthopsilosis* can cause damage in human tissues, the less virulent member of this complex being *C. metapsilosis* (Gácser et al., 2007). These latter two species are less prevalent than *C. parapsilosis*, but their incidence varies greatly depending on the location. It is important to note that not all of the microbiology laboratories make the distinction between the three species of this complex (Trofa et al., 2008). A recent study performed between January 2009 and February 2010 in Spain reported that 29.1% of fungemia cases were caused by *C. parapsilosis* s.l.. From these, 8.2% were *C. orthopsilosis* and 1.1% *C. metapsilosis* (Cantón et al., 2011). However, in a survey performed in hospitals in China, this scenario was inverted with *C. metapsilosis* accounting for 11.3% of all *C. parapsilosis* s.l. infections, and *C. orthopsilosis* only 3.7% (Xiao et al., 2015). In fact, geographical location could be an important factor in the distribution and incidence of opportunistic organisms indirectly, because medical procedures and the clinical environment differ across the world.

The elucidation of the evolutionary history of the *C. parapsilosis* complex has uncovered the existence of several hybrids related to *C. orthopsilosis* and *C. metapsilosis* (Table 1). *Candida parapsilosis* s.l. are diploid organisms and mating or meiosis has never been observed in this complex (Butler et al., 2009; Logue et al., 2005; Sai et al., 2011). *Candida parapsilosis* is described as a highly homozygous species, as recent whole genome analysis of various strains has confirmed (Butler et al., 2009; Pryszcz et al., 2013). In contrast, genomic analyses of *C. orthopsilosis* and *C. metapsilosis* provided a very different picture. Although the first genome sequence of a *C. orthopsilosis* strain depicted a highly homozygous sequence (Riccombeni et al., 2012), further genomic sequences revealed the existence of hybrids that had spread over distant geographical regions (Pryszcz et al., 2014; Schroder, 2016). The analysis of these hybrid genomes indicated a hybridization between the previously sequenced homozygous lineage and an unknown parent that showed 5% divergence at the nucleotide level (Pryszcz et al., 2014). Furthermore, it has been suggested that such hybrids have been formed at least four times independently, following independent hybridization events between the same parent lineages, and that the majority of *C. orthopsilosis* strains are hybrids (Schroder, 2016).

*Candida metapsilosis* genome is highly heterozygous and, contrary to what is described for *C. orthopsilosis*, all of the sequenced strains of this species are hybrids originated after a single hybridization event, and nothing is known about the parent species, except that they are 4.5% divergent (Pryszcz et al., 2015). The same authors hypothesize that, probably, the absence of the parent species in clinical samples is indicative of their inability to colonize or infect humans, and that the hybridization between these two non‐pathogenic lineages originated an opportunistic pathogen with worldwide distribution.

Although not as high as in *Cryptococcus*, the presence of a high number of hybrids in this clade is notable. This leads to the question of whether, for some reason, this clade has a greater propensity to generate hybrids than other *Candida* species. More studies are needed to confirm this idea, but in contrast to the case of *Cryptococcus* hybrids, the genomic aftermath of hybridization has been extensively studied in hybrids of *C. metapsilosis* and *C. orthopsilosis* (Pryszcz et al., 2014; Pryszcz et al., 2015; Schroder, 2016). An important observation regards the absence of differential loss of genomic material of the two parent species. In other studied hybrid species the stabilization of the genome generally drives to a disequilibrium between the proportion of genetic material of the parent lineages in the hybrid. This phenomenon has been observed, for example, in *S. pastorianus* genome, where sometimes one of the parent genomes is kept while the other one is almost entirely lost (reviewed in Morales and Dujon (Morales & Dujon, 2012)). In the more recent hybrid *Meyerozyma sorbitophila*, where 40.3% of the genome has undergone LOH, a parent inbalance is already apparent with 88.8% of the LOH sequence corresponding to the preferred parent sub‐genome (Louis et al., 2012). However, in the *C. parapsilosis* clade the proportion of the genetic material from each of the parent species in the LOH regions in the hybrids is close to 50% (Pryszcz et al., 2014; Pryszcz et al., 2015), showing that possibly these genomes do not present strong deleterious incompatibilities as in other clades. As mentioned before, LOH is an important indicator of genome shaping and stabilization. The number of LOH events in *C. metapsilosis* was shown to differ between strains, with some events being shared between all of them (Pryszcz et al., 2015). Even so, *C. metapsilosis* presents highly heterozygous regions in >50% of the genome, which is in contrast to the 17% of heterozygosity described for the MCO456 strain of *C. orthopsilosis (*Pryszcz et al., 2014 *;* Pryszcz et al., 2015 *)*. Owing to these differences, Pryszcz and colleagues proposed the existence of two possible scenarios: *C. metapsilosis* hybridization occurred more recently, or for some reason *C. orthopsilosis* genome is evolving faster (Pryszcz et al., 2015). The later work from Schroder and colleagues, showed that this MCO456 strain is associated with the oldest hybridization event of *C. orthopsilosis*, presenting one of the lowest heterozygosity values among the hybrid strains (Schroder, 2016). Indeed, within *C. orthopsilosis* it is possible to find strains with almost quadruple the heterozygous variants found in MCO456 (Schroder, 2016). Although these studies have been performed, several questions are still pending. For instance, it is as yet unknown whether the LOH events occur preferably at genomic regions encoding certain genes or functions, which could imply that these genes or functions are essential for the hybrid's survival. Answering this question would open the door to a better understanding not only of the genome stabilization phenomenon, but also of the special ability for the species of this complex to hybridize and the emergence of their ability to infect humans.

## Hybrids in other human fungal pathogens

The relevance of hybridization to human health is not exclusive to *Candida* or *Cryptococcus* clades. Gene exchange through hybridization was already reported in other species. Coccidioidomycosis is a pulmonary infection caused by the inhalation of *Coccidioides immitis* or *Coccidioides posadasii* spores, which are present in the soil and air. Although not being a dangerous disease in immunocompetent patients, it is estimated that 150 000 new cases of coccidioidomycosis occur annually in the USA, of which one‐third are fatal, being a special concern for immunocompromised persons (Odio et al., 2017). Genomic analyses of some populations from both species revealed the presence of a recent hybridization event (Neafsey et al., 2010), and more recently, the analysis of some genetic markers uncovered again the presence of hybrid organisms in these populations (Johnson et al., 2015). For instance, the genomic patterns of introgression between these two species revealed that at least 8% of the genes in *C. immitis* population may have been recently introgressed from *C. posadasii*, presenting an enrichment in genes associated with immune evasion and cell walls (Neafsey et al., 2010). Based on these results, the authors have proposed that these antigenic genes may present a selective advantage when introduced in the genome of the other species, which raises concern about the consequences of this hybridization for the virulence of these pathogenic fungi.

The same concern is appearing in relation to the filamentous fungi *Fusarium keratoplasticum*, the main causative agent of nosocomial infections associated with plumbing systems. *Fusarium* is responsible for skin lesions, pneumonia and disseminated infections in immunocompromised patients (Nucci & Anaissie, 2007), and strains of these fungi have been reported to present resistance to some anti‐fungal drugs (Azor et al., 2007; O'Donnell et al., 2008). Recent studies based on several genetic markers uncovered the presence of hybrid strains within this species, suggesting that this natural hybridization resulted in adaptation to the anthropogenic environments (Short et al., 2014; Short et al., 2013). Similar reports have been done for *Malassezia furfur*, a commensal fungus of the human skin, sometimes responsible for skin disorders, such as eczema or atopic dermatitis (Theelen et al., 2001). Recent advances in genomics and bioinformatics enabled the confirmation of a hybrid origin in some of its strains, where genes, usually present in one copy in this species, are present in two copies (Wu et al., 2015). A further phylogenetic analysis allowed confirmation that the hybrid strains were originated by hybridization events between two highly distant lineages of *M. furfur* (Wu et al., 2015). Therefore, the authors suggest that they should be regarded as intra‐species hybrids. Alternatively given the high distance between *M. furfur* lineages, if we consider *M. furfur* as a species complex, they could be considered as inter‐species hybrids (Wu et al., 2015).

Altogether, these findings show that hybridization is a transversal phenomenon in fungal pathogens, occurring in different clades (Figure 2). Moreover, the advent of genomics is allowing the discovery of such organisms, as well as study of their evolution and possible characteristics. Hence, it is possibly fair to assume that the number of known hybrid pathogens is far from the real total, and fungal hybridization is more frequent than previously thought. However, it is also important to note that the discovery of hybrids is just the first step towards understanding the evolution of hybrids in a clade and its possible role in the emergence of virulence. The examples described in this review are possibly only the tip of the iceberg. Of the five reported clades with hybrids, only two have been investigated to a certain degree, and constitute the most thoroughly studied cases. Yet studies on *Candida* and *Cryptococcus* hybrids have only superficially approached the mechanisms underlying hybrid genome evolution, and how they relate to phenotypic change, in particular with respect to their pathogenic behaviour.

## Concluding remarks and future prospects

Hybridization is a biological process responsible for the origin of new lineages or species with adaptation to new environments. The increasing identification of hybrid species with medical or economical importance shows that this evolutionary process has to be regarded as an important driver of evolution, especially in the fungal clade. Advances in medical procedures allowing the survival of immunocompromised patients, globalization and climate change are all factors probably underlying the increase in the number of hybrid opportunistic fungal pathogens. The trend is also likely to increase even further. Considering this, there is a need for investment in the development of new diagnostic tests in order to apply the optimal therapeutics. For this reason, it is important to understand the mechanisms underlying the hybridization events in fungi, especially those leading to the harmonious coexistence of two different genomes in one unique organism.

In this regard, the fields of genomics and bioinformatics are set to play a crucial role in future studies. In order to achieve this, the future will need to witness further developments of new programmes and tools especially directed to the assembly and analysis of fungal genomes, with a particular focus on highly heterozygous genomes. This should be regarded as an urgent task since good genomic sequences are the essential basis of achieving better knowledge on these pathogens. Short‐read paired‐end technologies are widely used for genome sequencing. However, difficulties in solving complex genome assemblies are leading to an integrated use of multiple technologies (Goodwin et al., 2016). Long‐read sequencing produces reads with several kilobases, which can be useful to solve regions where ambiguities are present (Goodwin et al., 2016), thus representing an important advance for hybrid genome assemblies. Even so, the higher error rates associated with long‐read sequencing are still a big concern. Although some works indicate that these are random errors, and therefore high coverage could overcome the problem, the fact is that these technologies can present an error rate up to 14% (Carneiro et al., 2012; Koren et al., 2012). Nevertheless, it is expected that new technological developments will progressively reduce error rates. Facing the problem of highly heterozygous assemblies, new pipelines such as ‘Redundans’ have recently been developed, contributing to an improvement in the quality of heterozygous genome assemblies (Pryszcz & Gabaldón, 2016). Nevertheless, there is still much room for improvement. For instance, ‘Redundans’ and other assembly programmes reconstruct chimeric reference genomes, comprising inter‐sparsed regions of the two sub‐genomes. Although subsequent mapping of genomic reads against this reference can distinguish homozygous from heterozygous blocks, in the absence of known parent species with a sequenced genome, it is not possible to reconstruct the origin of each of the sub‐genomes. There exist solutions for genome phasing, but these were specially developed for bacteria or mammals, and not for yeasts. This gap may be compromising the study of hybrid pathogens, and consequently, for example, the evaluation of the possibility that hybridization events are responsible for the emergence of new pathogens. Phasing hybrid genomes would help understand this problem, since in some cases, as mentioned before for *C. metapsilosis*, both parent lineages are unknown (Pryszcz et al., 2015), making it difficult to assess the consequences of such hybridization. Therefore, for a better understanding of these hybrid pathogens, new bioinformatics strategies have to be developed, which certainly will contribute to an improvement of our knowledge on hybridization in fungi, and consequently to the clarification of the consequences of this phenomenon for human health.

Genomic analyses can certainly increase our understanding of the evolution of hybrid pathogens, yet they need to be complemented with experimental functional analyses. Only a few studies have compared pathogenesis‐relevant phenotypes such as drug resistance, adherence or virulence among different hybrid strains and their homozygous parents. Only the comprehensive and carefully planned phenotyping of a large number of hybrid and non‐hybrid strains from which the genomes have been sequenced will help us to understand the genetic mechanisms underlying virulent traits in hybrids. Finally, transcriptome analysis performed with technologies such as RNAseq has the potential to reveal how transgressive phenotypes may be achieved in yeast hybrids and how hybridization can rewire transcriptional networks.

In conclusion, hybridization is a process suggested to be at the origin of new emerging pathogens (Hagen et al., 2015; Li et al., 2012; Pryszcz et al., 2015). There is still much to learn about these organisms, as current studies are only helping us to realize the potential role of hybridization in the emergence of pathogenesis, but we are as yet far from understanding this process. Although the real dimensions of this phenomenon are currently unknown, the currently described cases are likely to be only the tip of the iceberg. In addition, the emergence of hybrids with virulence potential may be increasing thanks to global trade and climate change. Finally, infections from hybrids may be more difficult to treat as they may be able to adapt faster owing to their intrinsically high genomic plasticity. Thus, pathogenic hybrids can represent a special concern for human health, and their study should be a matter of interest to us.

## Conflict of interest

The authors declare that there is no conflict of interest.
